# The Impact of Neuroimmune Alterations in Autism Spectrum Disorder

**DOI:** 10.3389/fpsyt.2015.00121

**Published:** 2015-09-09

**Authors:** Carmem Gottfried, Victorio Bambini-Junior, Fiona Francis, Rudimar Riesgo, Wilson Savino

**Affiliations:** ^1^Translational Research Group in Autism Spectrum Disorder (GETTEA), Federal University of Rio Grande do Sul, Porto Alegre, Brazil; ^2^Research Group in Neuroglial Plasticity, Department of Biochemistry, Federal University of Rio Grande do Sul, Porto Alegre, Brazil; ^3^Laboratory on Thymus Research, Oswaldo Cruz Institute, Oswaldo Cruz Foundation, Rio de Janeiro, Brazil; ^4^Sorbonne Université, Université Pierre et Marie Curie, Paris, France; ^5^INSERM UMR-S 839, Paris, France; ^6^Institut du Fer à Moulin, Paris, France; ^7^Child Neurology Unit, Clinical Hospital of Porto Alegre, Federal University of Rio Grande do Sul, Porto Alegre, Brazil

**Keywords:** autism, neuroimmune interactions, environmental risk factors, rodent models, valproic acid

## Abstract

Autism spectrum disorder (ASD) involves a complex interplay of both genetic and environmental risk factors, with immune alterations and synaptic connection deficiency in early life. In the past decade, studies of ASD have substantially increased, in both humans and animal models. Immunological imbalance (including autoimmunity) has been proposed as a major etiological component in ASD, taking into account increased levels of pro-inflammatory cytokines observed in postmortem brain from patients, as well as autoantibody production. Also, epidemiological studies have established a correlation of ASD with family history of autoimmune diseases; associations with major histocompatibility complex haplotypes and abnormal levels of immunological markers in the blood. Moreover, the use of animal models to study ASD is providing increasing information on the relationship between the immune system and the pathophysiology of ASD. Herein, we will discuss the accumulating literature for ASD, giving special attention to the relevant aspects of factors that may be related to the neuroimmune interface in the development of ASD, including changes in neuroplasticity.

## History of ASD Studies

The first use of the term “autistic” was in 1911, by the Swiss psychiatrist Eugen Bleuler (1857–1939), referring to the limitation of human relationships and the loss of contact with reality presented by patients with schizophrenia (1). The term was then adopted by the Austrian pediatrician Hans Asperger (1906–1980) working at the University Children’s Hospital-Vienna, referring to “autistic psychopaths.” Asperger was investigating a form of autism spectrum disorder (ASD) now known as Asperger syndrome and not widely recognized as a separate diagnosis until 1981. In 1943, the Austrian-American psychiatrist Leo Kanner (1894–1981) used the term “autistic disturbances of affective contact” to describe 11 children with behavior marked by difficulties in establishing affective and interpersonal contact (2). He reported a form distinct from other diseases, such as schizophrenia, and that seemed to affect patients from the beginning of their lives. In the following year, Hans Asperger described cases exhibiting some characteristics similar to autism, which included difficulty in social communication, but without cognitive loss. For further reading, see Ref. (3), in which Asperger’s 1944 manuscript was translated.

In 1980, the term “autism” was first inserted in the third edition of Diagnostic and Statistical Manual of Mental Disorders (DSM-III). In 1994, the fourth edition of the DSM included new criteria due to the need to identify subgroups of individuals with autism, for both practical purposes and research, considering the subdivisions: typical autism, pervasive developmental disorder not otherwise specified (PDD-NOS), and Asperger syndrome (4).

In the fifth edition, DSM considered instead of three domains of autism symptoms (social impairment, language/communication impairment, and repetitive/restricted behaviors), only two categories: (1) social communication impairment and (2) restricted interest/repetitive behaviors. Also, the new classification eliminated the previously separate subcategories into the broad term ASD (5–7). To simplify reading, the term “autism” or “ASD” will be used throughout the text representing the entire spectrum.

As a developmental disorder, ASD includes different degrees of severity and males are more affected than females by a ratio 5:1 approximately (8).

The number of cases in children increased by 23% between 2006 and 2008, reaching 1:88 children under 8 years diagnosed with any of the spectrum subtypes, and increased by 78% when the 2008 data were compared with the data for 2002. The overall prevalence of ASD for 2010 in the United States of America was 1:68 children aged 8 years and there is a clear growth in the number of identified cases (8). This can be due to advances in the knowledge of the symptoms associated with improvement in diagnostic criteria, as well as increase of environmental risk factors (drugs, pollutants, stress, etc.), especially during pregnancy, which may be related to changes in lifestyle of the society (8). In any case, this high prevalence indicates that the subject requires emergency measures due to the high economic, social, and family cost. The *Autism Speaks* organization estimates in the USA that the current costs of ASD reach US$137 billion per year, a number that has increased more than threefold since 2006.

## Clinical Approach and Molecular Phenotypes

There are two complementary issues in the clinical approach for autism. The first is the general management, including diagnosis and evaluation of the intensity level of eventual core behavioral symptoms (9). The second considers treatment options, such as psychopharmacotherapy and different types of non-medical treatments. It is important to consider that ASD symptoms usually change during the patient’s lifetime, and therefore, it is crucial for clinicians to be aware of age-related differences. Future perspectives in the treatment of ASD will probably include immunomodulation, stem cell therapy, and other approaches, after careful randomized controlled trials attesting the corresponding efficiency of these various strategies.

Although a number of definitions and improvements have been made in ASD, the etiological aspects remain unclear. The growing number of publications, especially in the last decade, leaves no doubt of the multifactorial aspect of the spectrum and indicates a complex interplay between genetic/environmental factors and the immune system, including stimulation of immune cells, generation of autoantibodies, cytokine/chemokine imbalance, and increased permeability of the blood–brain barrier (BBB) favoring leukocyte migration into the brain tissue (10).

In addition to clinical knowledge related to ASD, intense efforts have been directed toward identifying genes that specifically cause or increase the risk of developing autism, through both large genome-wide association studies and investigation of new candidate genes (11–16). It is estimated that 400–1000 genes may be related to ASD and large-scale studies in ASD and respective families have allowed the identification of candidate genes that may be related to the development of this disorder. Single-gene polymorphisms have been associated with ASD (17, 18), including those affecting contactin-associated protein like 2 (*CNTNAP2*); SH3 and multiple ankyrin repeat domains 3 (SHANK3); neuroligin 3 (NLGN3/4); copy-number variations and chromosomal abnormalities, such as the 15q11–q13 duplication and 16p11.2 deletions or duplications.

Other ASD candidate genes include forkhead box P2 (FOXP2); suppression of tumorigenicity 7 (ST7); IMP2 inner mitochondrial membrane peptidase-like (IMMP2L); reelin (RELN) at 7q22–q33, gamma-amino butyric acid (GABA)A receptor subunit; and ubiquitin-protein ligase E3A (UBE3A) on chromosome 15q11–q13 (19).

Table 1 illustrates polymorphisms with correlation to gut–brain axis abnormalities. The communication between these two systems needs to be further studied in order to identify possible key elements involved in ASD symptomatology. We mention a few examples as follows. The protein MET is a pleiotropic tyrosine-kinase receptor that functions in both brain development and gastrointestinal repair. An important functional variant (rs1858830 allele “C”) of the gene encoding this protein has been demonstrated to be strongly associated with autism, as seen in a group of patients that also presented gastrointestinal disturbances (20) and altered immune response (21). Also, it was demonstrated that MET protein levels were significantly decreased in the cerebral cortex from ASD cases, compared to healthy controls (22), suggesting a dysregulation of signaling that may contribute to altered neural circuit formation and function. As the MET receptor plays important function in regulating immune responsiveness (21), it is conceivable that it has a simultaneous influence upon both brain development and gastrointestinal function.

**Table 1:** **Selected genes altered in ASD, correlated with immune function**.

Gene	Protein	Function
*MET*	Receptor tyrosine kinase (MET)	
*PTEN*	Phosphatase and tensin homolog (PTEN)	
*TSC1*	Tuberous sclerosis complex-1 (TSC1)	Promote IL-12 increase and M2-macrophage conversion
*TSC2*	Tuberous sclerosis complex-2 (TSC2)	
*HLA-DRB4*	Major histocompatibility complex type II (MHC-II)	
*MIF*	Macrophage migration inhibitory factor (MIF)	Guide and control of immune response
*C4B*	Complement component 4B (C4B)	

Overall, these data indicate molecular phenotypes, genetic risk factors, and gastrointestinal abnormalities, with the gut–brain axis. This hypothesis emerges from the observation that MET expression is decreased in temporal cortex from postmortem ASD brains and that the endogenous MET ligand, hepatocyte growth factor (HGF) is decreased in the gastrointestinal tract from ASD patients (17).

In a second vein, specific haplotypes related to the polymorphism of the *SLC6A4* serotonin transporter (SERT) gene correlate with hyperfunctioning of serotonin transporter SERT in brain, in circulating platelets, and in enterocytes (17), further indicating interconnections between genetic risk factors for autism and gastrointestinal abnormalities. The *SLC64A* gene is found on chromosome 17q11–12 and encodes one of the SERT genes. The 5-hydroxytryptamine-transporter length polymorphism (5HTTLPR) of the *SLC64A* gene has been considered to be associated with abnormalities seen in serotonin transporter binding in ASD (17, 23, 24). Serotonin receptors have also been found in the gut mucous layer (25), indicating possible implications in ASD since drugs that alter serotonin levels are taken orally.

In future studies, it will be important to improve the understanding of the relationships between genetic variation and phenotype. In fact, the wide diversity of core features in ASD and a varied occurrence of comorbidities make diagnostic procedure and clinical management of the patient more difficult, presenting a complex range of brain alterations with important changes in the frontal cortex.

It should be pointed out that, in addition to genetic alterations, environmental risk factors (such as infections, and drug use) during key periods of embryonic/fetal development may be associated with triggering ASD (26). It was demonstrated that modeling a situation of maternal infection (by maternal immune activation, MIA) in mice leads to permanent immune dysregulation in the progeny animals, together with autistic-like symptoms.

## Cortical Connectivity Dysfunction in ASD

Although a consensus concerning structural and functional abnormalities in ASD remains difficult, a number of studies on these topics bring together important data, as shown in Table 2. Several abnormalities have been identified, which may have a relationship with neuroimmune changes during development. These include subtle defects in cortical architecture, aggravated perhaps by perturbed critical period activity-dependent remodeling of the network. Such changes lead to white matter defects and connectivity problems, which can, in some cases, be linked to behavioral abnormalities, as discussed below.

**Table 2:** **Anatomical studies of brains from individual with ASD**.

Phenotype	Brain area	Method	Studied	Age (autism)	Sex	Reference
Macrocephaly	Head	Head circumference	*n* = 208 probands, *n* = 147 parents, *n* = 149 siblings, and other controls	9.7 ± 5.4 years (3–47 years)	5.9M:1F	(27)
Neuron number	DL-PFC and M-PFC	Postmortem	*n* = 7 autistic, *n* = 6 control	2–16 years	Male	(28)
WM volume	Head	MRI	41 boys (13 autistic, 14 DLD, 14 normal control); 22 girls (7 DLD, 15 normal controls)	9.0 0.9 years (autistic), 8.2 1.6 years (DLD), and 9.1 1.2 years (controls)	Males and females	(29)
GM volume, Gyral thickness	Head-temporal and parietal lobes affected	MRI	*n* = 17 autism, *n* = 14 controls	8–12 years	Male	(30)
PV interneurons	DLPFC	Postmortem	*n* = 2 ASD *n* = 2 matched controls for age, sex, and hemisphere	30–44 years	Male	(31)
Minicolumns	Prefrontal (area 9) and Temporal (areas 21, 22) lobe	Postmortem	*n* = 9 autism brains and *n* = 4 and 5 controls	5–28 years	–	(32)
Neuron migration disorders	Brain	Postmortem	*n* = 13 autism, *n* = 14 controls	4–62 years	9 males 4 females	(33)
Cell density cortical layers	ACC	Postmortem	*n* = 9 autism brains and controls			(34)
Cortical layers	PCC FFG	Postmortem	*n* = 8–9 autism, *n* = 7–8 control	19–54 years (PCC), 14–32 years (FFG)	Male	(35)
Dendritic spines	Frontal, temporal and parietal (layer II), layer V (temporal)	Postmortem Golgi	*n* = 10 autism; *n* = 10 and 5 controls	10–45 years	Males	(36)
Gray-white matter boundary	STG, DL-frontal, and DL-parietal		*n* = 8 ASD, *n* = 8 control	10–45 years	Males	(37)
Axons	ACC, OFC, LPFC	Postmortem	*n* = 5 autism, *n* = 4 control	30–44 years	4 male, 1 female	(38)
Corpus callosum	CC	MRI	*n* = 253 autism, *n* = 250	Meta-analysis (10 studies)	Male >74%	(39)
Corpus callosum	CC	MRI	*n* = 17 autism, *n* = 17 control	16–54 years	Males and females (3)	(40)
Brain development	White matter	DTI (prospective, longitudinal)	*n* = 28 ASD, *n* = 64 control	6–24 months	Males and females	(41)
Brain	White matter (CC)	DTI (prospective, longitudinal)	*n* = 100 ASD, *n* = 56 controls	3–41 years	Males	(42)
Brain	White matter (CC)	DTI	*n* = 43 autism (or PDD, or ASD); *n* = 34 controls	7–33 years	Males	(43)
Brain	White matter and activation (ACC)	DTI and fMRI	*n* = 12 ASD (autism), PDD or Asperger; *n* = 14 control (6F)	20–40 years	Males and females (2)	(44)
Brain	White matter (arcuate)	DTI	*n* = 13 autism; *n* = 13 siblings, *n* = 11 controls	6–13 years	Males and females (2)	(45)
Brain	White matter (several areas)	DTI	*n* = 7 autism; *n* = 7 controls	11–18 years	Males and females (1)	(46)
Brain	Theory of mind areas	fMRI	*n* = 12 high functioning autism; *n* = 12 control (6F)	15–35 years	Males and females (2)	(47)
Brain	Several areas	MRI/DTI	*n* = 18 autism; *n* = 18 control	6–12 years	Males and females (2)	(48)
Brain	Language and spatial	fMRI	*n* = 12 autism; *n* = 13 control	Mean 22.5 years	Males and females (1)	(47)
Brain	Working memory face recognition	fMRI	*n* = 11 high functioning autism; *n* = 11 controls	24.5 ± 10.2 years	Males	(49)
Brain	Reading comprehension	fMRI	*n* = 17 high functioning autism; *n* = 17 controls	–	–	(50)
Brain	Resting state	fMRI	*n* = 13 (6 autism; 6 Asperger; 1 PDD); *n* = 12 control	15–52 years	Males	(51)
Brain	Resting state	fMRI	*n* = 16 high functioning (6 autism, 2 Asperger; 8 PDD); *n* = 15 controls	13–17 years	Males and females (2)	(52)
Brain	Executive function (Tower of London task), CC size	fMRI	*n* = 18 high functioning autism; *n* = 18 controls	27.1 ± 11.9 years	Males and females (1)	(50)
Brain	Resting state	fMRI	*n* = 12 high functioning; *n* = 12 control	26 ± 5.93 years	Males and females (1)	(53)
Brain	Source recognition task	fMRI	*n* = 10 ASD 6 autism, 4 Asperger; *n* = 10 control	14–43 years	Males	(54)
Brain	Face processing	fMRI	*n* = 19 high functioning (8 autism, 9 Asperger, 2 PDD) and *n* = 21 control	23.5 ± 7.8 years	–	(55)

As previously mentioned, structural abnormalities are likely to be developmental in origin but may have diverse causes. Yet, before entering into this issue, it seemed worthwhile to describe normal cortical development (Box 1), also described in Ref. (56).

Box 1 **General features of normal cortical development.**

During early steps of cortical development, stem cells and progenitor cells divide in zones close to the cerebral ventricles before giving rise to neurons, which migrate long distances to reach the developing cortical plate. Future principal and inhibitory neurons are derived mainly from dorsal and ventral telencephalic regions, respectively. Critically timed neuronal activity is essential for circuit development, both intrinsic activity and sensory derived, affecting synaptogenesis and remodeling. Synaptic pruning removes unused and unwanted connections to refine the synaptic patterns. Timing is critical and activity-dependent processes contribute to spine turnover and maturation (57). Excitatory synapses are generally formed first, followed by inhibitory synapses. The temporal regulation of synaptogenesis is likely to be highly regulated for a correct excitatory: inhibitory balance. Myelination of mature neurons is another critical process ensuring correct functional connectivity in a timely fashion between neurons. In the primate, it has been shown that cortical areas take different amounts of time to form (58).

Environmental risk factors acting during cortical development (*in utero* effects related to maternal infections, stress, other agents, such as pharmaceutics, alcohol and drugs of abuse, and postnatal experience-dependent activities), can, hence, have heterogeneous influences on the formation of cortical areas. For example, maternal autoimmunity, infection during pregnancy, maternal age and obesity, gestational diabetes, and the presence of maternal *MET* variant rs1858830 “C” allele have been associated with the triggering of ASD through maternal immune activation, which could manifests as changes in the maternal peripheral cytokine milieu, generation of immunoglobulin G (IgG) and autoantibodies reactive to fetal brain proteins in different areas, such as frontal cortex (59).

Increased brain size (“macrocephaly”) in the first years of life is now firmly associated with ASD (60, 61). This may have its origins in increased numbers of neurons in some brain areas (as the prefrontal) compared to normal individuals (28), or more prominently in increased cell size (soma, axonal tracts, and dendrites), or in combination of both, in localized regions. Accordingly, differences are observed in ASD patients in gray or white matter volumes, both identified in MRI studies (62, 63). These increased volumes in ASD are associated with aberrant connectivity, which may combine over and under-connectivity. As mentioned below, structural and functional data revealed a connectivity disturbance, affecting frontal, fronto-temporal, fronto-limbic, fronto-parietal, and inter-hemispheric connections (31, 64).

Concerning potential gray matter abnormalities during childhood, in postmortem studies, 79 and 29% more neurons were identified in dorsolateral and medial prefrontal regions, respectively, in ASD (28). Furthermore, subtle defects in the radial organization and local densities of neurons (“minicolumns”) in different cortical areas, including the frontal cortex have been identified in brains from both ASD children and adults (65, 66), reviewed by Zikopoulos and Barbas (31). Occasionally, nodular subependymal heterotopia has been identified in ASD (33), suggesting local progenitor or neuronal migration abnormalities, although this may be rare. In the adult, increased numbers of neurons are not obvious (31), although minicolumn changes, with subtle abnormal lamination have been identified occasionally in certain areas (35, 67).

Increased dendritic spine densities have also been observed notably in layer 2 of lateral prefrontal association areas (36). Parvalbumin positive interneurons have been shown in postmortem studies to be less numerous in dorsolateral prefrontal regions (31). Blurred white and gray matter boundaries are also regularly observed in ASD (37, 65), and this has been suggested in other situations (e.g., schizophrenia) to be due to an excess of interstitial neurons in the white matter, which may have their origin in the subplate (68). Overall, several neuronal density and distribution alterations, localized to certain areas, are associated with ASD.

A number of changes in brain seem to be related to late prenatal or early postnatal periods. Transiently enlarged white matter volumes (related to abnormal axonal tracts) have been documented in ASD infants exhibiting enlarged head circumferences. White matter volumes in these individuals then increase less slowly during childhood compared to control individuals [reviewed by Cassina et al. (69)]. Axonal tracts have been studied by confocal and electron microscopy in postmortem tissue (38), showing fewer large axons in the deep white matter of the anterior cingulate cortex (likely representing long-range cortico-cortical connections), a higher proportion of branched axons of medium caliber, and a significantly increased density of thinner branched, axons in the superficial white matter (likely connecting nearby areas). Other neuroimaging studies have shown reductions in the strength of long-distance connections, e.g., sensory input to prefrontal cortex and inter-hemispheric connections (40, 43, 70–72). Such defects would be expected to have quite severe network effects.

Travers et al. (73) (and references therein) summarize and compare 48 peer-reviewed diffusion tensor imaging (DTI) studies. Preliminary findings suggest that developmental trajectories of fractional anisotropy in ASD infants are also different from controls, and may mimic the accelerated brain volume phenotype (41, 73). Despite small sample sizes, the corpus callosum was found in several DTI studies to be reduced in volume [Ref. (43), see Figure 3 of Ref. (73)], and in one study the authors further found this result to be specific to patients who did not have macrocephaly (61). Interestingly and *vice versa*, callosal agenesis is also associated with autism-like symptoms. Concerning microstructure, fractional anisotropy was found reduced in anterior regions or across the length of the corpus callosum in multiple studies (42, 43, 73). This may be due to reduced myelination or larger axon diameter or reduced density. In some studies, this finding was associated with reduced performance IQ and reduced callosal volume (43). Differences were observed in ASD patients, concerning the cingulum bundles, which are primary inter-hemispheric-association pathways associated with executive function, connecting the medial cingulate cortex with temporal lobe structures, such as the hippocampus, consistent macrostructural, and reduced fractional anisotropy (44). Relatively ­concordant results of decreased fractional anisotropy were obtained at the beginning of the arcuate fasciculus (although heterogeneous results were obtained for the whole tract) in the region of the temporo-parietal junction and superior temporal gray matter (45, 46, 73). This latter region is associated with social perception, and gray matter structure and functional connectivity differences have also previously been identified (47). For the moment, relationships between DTI measures and ASD symptoms remain only preliminary and future work with defined patient groups will deepen these correlations [see also Ref. (44)].

Studies using DTI also show differences in the cerebellar fibers that connect to various brain regions, demonstrating altered cerebellar feedback projections in ASD (74). In addition, neuropathological studies have also reported a decrease in Purkinje cell density in the cerebellum of ASD patients indicating that this abnormality may contribute to selected clinical features of the autism phenotype (75).

Functional magnetic resonance imaging (fMRI) studies are being used to assess synchronous activated and deactivated brain regions during cognitive tasks and in resting states in ASD patients [reviewed by Rathinam et al. (76)]. It appears that the most consistent functional results refers to a decreased connectivity between frontal and more posterior brain regions (in high-functioning patients), performing a variety of tasks. These include task integrating language comprehension (frontal) and spatial processing (parietal) (77); in working memory tasks related to face recognition [involving frontal executive and occipito-temporal fusiform gyrus regions (49)], and in reading comprehension requiring language comprehension and working memory (50). Similarly, frontal-posterior under-connectivity has also been found in studies of patients at rest, revealing hence spontaneous brain activity connections (51, 52, 78). These resting state studies suggest that abnormal connectivity may already exist in patient brains, not specifically related to different tasks, and perhaps indicating a structural basis for some differences, as suggested above. There is, however, some heterogeneity in other fMRI results, since some tasks in some patients have also shown frontal-posterior over-connectivity (79), fronto-frontal, or posterio-posterior over-connectivity in the resting state (53). Analyzing connections with other brain regions, e.g., subcortico-cortico, has also in several cases revealed over-connectivity [e.g., in task-independent tasks, Ref. (72) and references therein], or under-connectivity (55). A variety of other brain regions have been analyzed contributing to the variability of the results obtained [Ref. (80) and references therein]. In addition, transcranial ultrasonography may be a useful screening technique for children at potential risk of ASD, providing rapid, non-invasive evaluation of extra-axial fluid and cortical lesions (81). Further work, potentially involving new methods, may help to clarify under- or over-connectivity in different brain regions.

Whether or not these changes are related to neuroimmune interactions is a completely open field of investigation. In particular, it should be helpful to perform correlation studies between the above described changes with specific immune activation states, such as infections.

## Crosstalk between the CNS and the Immune System in ASD

The crosstalk involving the immune and nervous systems encompasses a complex and intricate pathway of signals with extensive communication between them in health and disease (82, 83). Cytokines and chemokines modulate brain function, as well as systemic and CNS responses to infection, injury, and inflammation (84). In fact, cytokines, such as TNF-α, IL-1β, IL-6, and TGF-β family, are able to modulate neuronal activity (85) and IL-6 promotes oligodendrocyte survival (86).

Pro-inflammatory cytokines, including interleukin (IL)-1, IL-6, IL-12, interferon-γ (IFN-γ), and tumor necrosis factor α (TNF-α), are involved in CNS pleiotropic effects during neurodevelopment (87) and have been extensively studied in patients with ASD. In a pioneer work indicating immune dysfunction in ASD (88), cell-mediated immune response was assessed *in vitro* by phytohemagglutinin (PHA) stimulation in lymphocyte cultures from 12 children with ASD and 13 control subjects: the ASD against neural antigens, produced by the mother during pregnancy (89–92), and that may induce changes in neural development and plasticity in the developing embryo/fetus.

Anti-double-stranded DNA antibodies and anti-nuclear antibodies were measured in the sera of 100 autistic children, aged between 4 and 11 years, in comparison to 100 healthy-matched children (93). In this study, the authors found increased levels of anti-double-strand DNA (34%) or anti-nuclear antibodies (25%) in ASD children. Furthermore, meta-analysis of data reported in patients with ASD clearly revealed alterations in different cytokines, both in plasma and in brain, as seen in Table 3.

**Table 3:** **Altered cytokines in autism spectrum disorder (ASD)**.

Cytokines	Level compared to control group	Source	Evaluated subjects	Reference
**INTERLEUKINS**				
IL-1β	↑	Plasma	Children with ASD	(94)
	↑	Plasma	Children with ASD	(95)
	↑	Plasma	Adults with severe ASD	(96)
	↑	Blood cells	Children with ASD	(97)
	↑ (TLR2 or TLR4 stimulation)	Blood cells	Children with ASD	(98)
	↓ (TLR-9 stimulation)	Blood cells	Children with ASD	(98)

IL-6	↑	Plasma	Children with ASD	(94)
	↑	Plasma	Adults with severe autism	(96)
	↑	Blood cells	Children with ASD	(97)
	↑ (TLR2 or TLR4 stimulation)	Blood cells	Children with ASD	(98)
	↓ (TLR-9 stimulation)	Blood cells	Children with ASD	(98)
	↑	Lymphoblasts	Children with ASD	(99)
	↑	Cerebellum (postmortem)	Children with ASD	(100)
	↑	Brain (postmortem)	ASD subjects (children and adults)	(101)
	↑	Brain (postmortem)	ASD subjects (children and adults)	(102)

IL-12 P40	↑	Plasma	Children with ASD	(94)
**CHEMOKINES**				
CCL2	↑	Brain (postmortem)	ASD subjects (children and adults)	(101)
		Plasma	Children with ASD	(94)
**TUMOR NECROSIS FACTOR**				
TNF-α	↑	CSF	Children with ASD	(94)
		Brain (postmortem)	Children with ASD	(103)
**INTERFERON**				
IFN-γ		Serum (mid-gestational)	Mothers giving birth to child with ASD	(6)
	↑	Whole blood and serum	Children with ASD	(104)
		Brain (postmortem)	ASD subjects (children and adults)	(103)
**GROWTH FACTORS**				
TGF-β1	↓	Plasma	Children with ASD (Lower levels correlated with more severe behavioral scores)	(105)
	↓	Serum	Adults with ASD	(106)
BDNF	↑	Brain (postmortem)	ASD subjects (children and adults)	(101)
	↑	Plasma	Children with ASD	(94)

*IK, interleukin; IFN, interferon; TGF, transforming growth factor; TLR, toll-like receptors*.

Also, although ASD patients present reduced amounts of total IgM and IgG immunoglobulins contents, they exhibit increased levels of antibodies against various proteins expressed in the nervous tissue, e.g., serotonin receptors, myelin basic protein, heat shock protein, and glial fibrillary acidic protein (GFAP) (107, 108). Recently, the presence of autoantibodies against human neuronal progenitor cells (NPCs) was assayed in sera from children with ASD (109). Immunoreactivity against multiple NPC proteins of molecular sizes ranging from 55 to 210 kDa was found in the ASD group, significantly differing from control individuals. This is in keeping with the fact that in the mouse model of autism following maternal immune activation triggered by poly(I:C)-injection, offspring exhibited a reduction of 50% in the numbers of regulatory T lymphocytes (CD4^+^Foxp3^+^CD25^+^) in the spleen (110), indicating a dysfunction in the regulation of the immune response in autism.

As mentioned above, studies in animal models indicate that maternal immune activation leads to autistic-like behavioral patterns in the offspring (111, 112). In addition to B and T cell abnormalities, changes in the innate immune response have been reported. Using *in vitro* experiments, it was demonstrated that ASD individuals have a reduced capacity of natural killer (NK) cells to kill K562 target cells (an immortalized myelogenous leukemia cell line) (113). Thus, it is likely that an aberrant group showed impaired lymphocyte PHA-induced proliferation when compared to control subjects.

In the following years, the hypothesis of autoimmunity involving the CNS was postulated as a key issue in the pathogenesis of autism and various clinical studies indicated a link between dysfunctional immune activity and ASD, including maternal immune abnormalities during early pregnancy (10, 114) and increased incidence of familial autoimmunity (115). Additionally, autoimmunity triggered by viral or bacterial infections has been considered as risk factor to ASD development (87, 116, 117). It has also been demonstrated in humans that family history of autoimmune disorders is more common in families of children with ASD (118). In addition, immune-mediated disorders during pregnancy, such as allergy and psoriasis, are more frequent in mothers of children with ASD compared with mothers of children with typical development (119).

Yet, the biological mechanism(s) of maternal immune dysfunction that could be involved in triggering ASD remain(s) unclear. One possibility involves the generation of antibodies activity of these components of innate immunity may also contribute to atypical immune activity seen in patients with ASD.

Moreover, increased numbers of circulating monocytes, important precursors for macrophages, dendritic, and microglial cells, have been observed in the blood and in the postmortem brain tissue from ASD individuals, associated with the presence of perivascular macrophages (101, 120). Furthermore, analysis of cytokine serum levels in children with ASD revealed a representative profile of myeloid cell activation, with increased production of IL-14, IL-12p40, TNF-α, IL-1β, and IL-6 (94–97, 121). Also, increased level of TNF-α was found in cerebrospinal fluid of children with ASD (122).

In respect to caspases, a group of cysteinyl aspartate-specific proteases involved in apoptosis and several other cell functions, it has been shown that the activation of some members of the caspase family contributes to the differentiation of monocytes into macrophages, in the absence of cell death (123). Interestingly, the mRNA levels for caspases 1–5, 7, and 12 were significantly increased in ASD patients as compared to healthy subjects, suggesting a role of the caspase pathway in ASD clinical outcome and as potential diagnostic and/or as therapeutic tools (124). These studies will hopefully provide new insights in the mechanisms of caspase activation and abnormal differentiation of monocytes into macrophages in ASD.

Considering that monocytes are key elements for the immune response, these alterations may result in long-term immune alterations in ASD children, with adverse neuroimmune interactions, ultimately contributing to the ASD pathophysiology. Also, it was found increased expression levels of pro-inflammatory cytokines TNF-alpha and IL-6, and decreased Bcl2 expression in lymphoblasts (99) and decreased levels of TGF-β in plasma (105) and in serum (106) of autistic subjects.

Moreover, considering that increased levels of anti- and pro-inflammatory cytokines have been observed in ASD individuals (6), it is conceivable that cytokines are also involved in the pathophysiology of ASD.

Taking into account, the environmental *in utero* influence in triggering ASD changes in oxidative stress responses may also correlate with activation of the hypothalamic–pituitary–­adrenal (HPA) axis. Upon activation, the hypothalamus secretes corticotropin-releasing hormone (CRH), stimulating the anterior pituitary gland to secrete adrenocorticotropic hormone (ACTH), which in turn stimulates the cortex of the adrenal glands to release glucocorticoid, which plays an important role in adaptive responses (125), including immunosuppression. This response can signal to the organism under stressful events, such as environmental adverse factors *in utero*. In fact, patients with ASD present elevated blood levels of nitric oxide (NO), nitrites, and nitrates (126). These molecules might increase the permeability of BBB and intestinal permeability, as commonly found in autism (127). Furthermore, ASD patients have diminished antioxidant systems in plasma, including decreased amounts of glutathione (GSH), vitamins (A, C, and E), and antioxidant enzymes (superoxide dismutase and glutathione peroxidase) (128–130). The increase in oxidative stress can potentially induce dysfunction in the immune system, plasticity and function of the thymus and stimulate neuroinflammatory infiltrates. Potentially, this set of dysfunctions may be associated with the behavioral abnormalities, gastrointestinal disorders, and sleep disturbances present in autism. In an animal model of ASD induced by prenatal exposure to valproic acid (VPA), a reduced thymus size was observed in the VPA group, compared to the control animals (131), indicating that T-cell development can also be affected in autism, and may be at the origin of both T and B cell dysfunctions seen in ASD, including neuroinflammation.

One important point is that, although the well accepted fact that the CNS undergoes constant immune surveillance that takes place within the meningeal compartment (132), the real mechanism(s) that guide(s) the entrance and exit of immune cells from the CNS remains to be demonstrated. Recently, an interesting investigation revealed the presence of structures with functional lymphatic vessels lining the dural sinuses, in a place difficult to visualize and actually so far ignored. These structures present characteristics of lymphatic endothelial cells and are able to carry both fluid and immune cells from and into the cerebrospinal fluid. Importantly, these structures are connected to the deep cervical lymph nodes (132). From this view, it is clear that a new and important window of investigation starts, in the search for possible link(s) connecting triggering of ASD to immune system impairment and *vice versa*.

## Evidence for Neuroimmune Interactions in ASD

The intercommunication between the brain and blood systems is followed by integrative exchanges, and the BBB permeability is variable, depending on the vessel type (artery, capillary, or vein) (8). During development, neurons, astrocytes, oligodendrocytes, and microglia intercommunicate in a paracrine/autocrine manner (133), withstand endocrine and immune systems influences, particularly during pregnancy, which can impair functions of the nervous system. Microglial cells in turn act as surveillance systems, with the capacity to respond phenotypically with varying degrees of activation to fluctuations in microenvironment stimuli or to transient or chronic damage, reaching the phagocytic state in the event of cell death (134). These cells also present dynamic movements or projections able to detect irregularities in neural microenvironments, in both intra and extracellular milieu and can increase in number by proliferation or through the entrance of macrophages into the brain (135).

In this vein, it was demonstrated by analysis of postmortem brain tissue that individuals with autism have an increased number of activated microglial cells (136).

**Figure 1:**
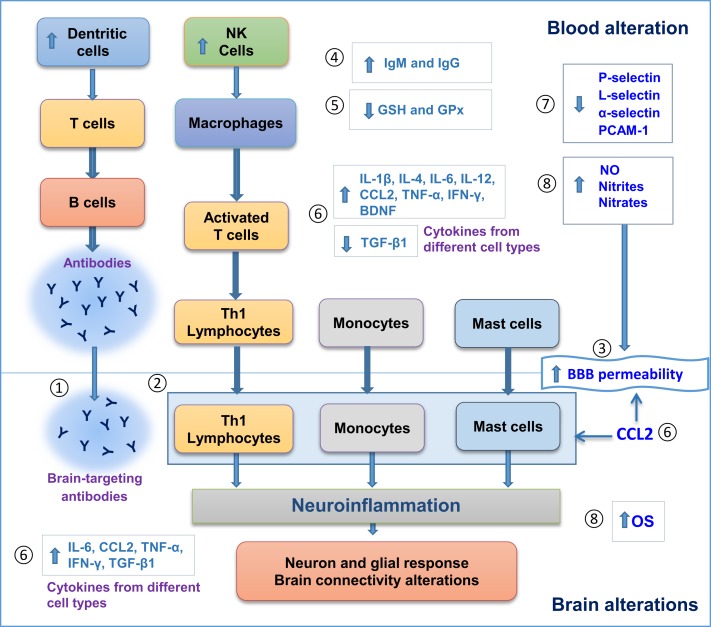
**Evidence for neuroimmune interactions in autism spectrum disorder (ASD)**. Blood and postmortem brain alterations in individuals with ASD. (1) Antibody production in blood against brain antigens. (2) Brain cell infiltration of Th1 lymphocytes, monocytes and mast cells. (3) Increase in blood brain barrier (BBB) permeability. (4) Increase in IgG and IgM levels. (5) Less antioxidant defenses. (6) Changes in cytokine levels. (7) Decrease in cell adhesion molecules, such as Selectins and PCAM-1. 8. Increase in oxidative stress. All these alterations can promote neuroinflammation, followed by neuron–glial response and brain connectivity dysfunction that ultimately can influence behavioral features in ASD. GSH, glutathione; GPx, glutathione peroxidase; NO, nitric oxide; Th, T-helper; OS, oxidative stress; CCL2, C–C motif chemokine 2.

Figure 1 illustrates alterations found in both blood and postmortem brains of patients with ASD, including blood/brain cell activation, autoantibody production, and alterations in levels of different molecules that can modify cell signaling, brain response, and BBB permeability. The associated neuroinflammatory process does support the hypothesis of neuroimmune interactions in the pathogenesis of ASD.

The analysis of postmortem brains from ASD individuals indicates changes in synaptic organization, dendritic arborization, neurotransmission (i.e., GABAergic, serotonergic, and glutamatergic pathways), and glial cells. Accordingly, recent studies suggested an important role for astrocytes and microglial cells in ASD, with alterations in GFAP expression (137), and increases of pro-inflammatory cytokines (6).

Molecules secreted by the brain’s immune system may influence neurodevelopment. As already mentioned above, individuals with autism have a marked neuroinflammation, with microglial activation and increased NO, as well as production of chemokines and pro-inflammatory cytokines (6, 101). There is evidence that an increase of TNF-α is associated with stereotypic behaviors similar to those found in individuals with autism (138). Moreover, soluble cytokine receptors that are normally present in blood can regulate peripheral cytokine and lymphoid activity (139–141). Further elucidation and characterization of the molecular pathways that mediate soluble cytokine receptor signaling in ASD will promote new strategies for therapeutic interventions.

In addition, as demonstrated in Table 1, the genes *MET*, *PTEN*, *TSC1*, and *TSC2*, for example, encode proteins related to the phosphoinositide3-kinase (PI3K) pathway, which plays an important role in suppressing the production of the pro-inflammatory cytokine IL-12 (142). MET is important to the developing brain, particularly to the neocortex and cerebellum in two regions frequently compromised in autism (22). Also, alteration in this gene can be correlated with increased immune response, involving cytokine expression (21) and regulation by small RNAs (miRNA) (143), which are presently known to be associated with the immunological response, such as lymphocytic phenotypes or key points during hematopoiesis (144). In ASD, various miRNA are altered in the blood, providing new clues in the search for new molecular targets in the study of autism (145–147). One is miR-132, altered in both autism and schizophrenia, and that can participate in brain plasticity, connectivity, and regulation of immune responses (145).

Another area implicated in autism is the cerebellum, and immunological studies indicate increased levels of IL-6 in the cerebellum of ASD subjects, stimulating the formation of granule cell excitatory synapses, without affecting inhibitory synapses (100).

A relevant point to be considered in the neuroimmune interactions occurring in autism is the fact that the intestinal mucosa of children with autism has a higher frequency of TNF-α^+^ T cells and lower frequency of IL-10^+^ T cells (148, 149). These studies indicate that such lymphocytes assume a pro-inflammatory profile, which corroborates with the increased levels of pro-inflammatory cytokines found in plasma and brain of patients with ASD.

Another important issue is the strong association between autism and allergic response involving mast cells, which correlates with various cellular processes, including allergic reactions enteric nervous system (ENS) (87, 150).

Increased plasma levels of IgG4 in children with ASD were also observed (151). These changes may be linked to changes in BBB permeability and also may influence neural plasticity and function, resulting in impairment in social interaction, communication, and behavior (87).

It is also important to consider cell adhesion molecules (CAM), which are present in endothelial cells, promoting a direct and selective interaction between blood cells and the cerebral endothelium (152). It is well known that CAMs play an important role in mediating the passage of T cells through endothelial barriers (153). These data indicate that the modulation of immune cell entry into the brain from patients with autism might also be a potential therapeutic target.

Working with the animal model of ASD induced by prenatal exposure of VPA, we recently demonstrated that the treatment of pregnant females with the antioxidant and anti-inflammatory resveratrol (RSV), before and after VPA exposure, prevented all behavioral impairments observed in the offspring (154). This is a naturally occurring phytochemical that was detected in 1963 in the dried roots of *Polygonum cuspidatum* (Itadori tea) and has been proposed as a pharmacological tool for neuroprotection against neuronal injury, including age-associated chronic diseases (155), ischemic brain damage (156), and cerebral models of stroke (157). For a systematic review and recommendations on the use of RSV, read (158).

Since similar alterations are also observed in the animal model induced by VPA (131, 159–161) and RSV exerts anti-inflammatory effects (158), future studies will be relevant to evaluate the influence of RSV in the immune system, particular in the ASD context. There is evidence for RSV use to establish immunological tolerance during treatment of autoimmune diseases that ablate or suppress the immune system. Specifically, RSV effect on tolerance is likely to be in the induction of Foxp3^+^ T cells and IL-10 expression, which are critical to development of T cells that are protective against autoimmune diseases, such as multiple sclerosis (162). In addition, the administration of RSV to mice developing experimental autoimmune encephalomyelitis – an animal model of human multiple sclerosis – increases expression of IL-10 and Foxp3 in T cells, the animal model of multiple sclerosis (163). In order to advance the knowledge related ASD development, it is important to also evaluate intracellular targets of VPA and RSV to clarify molecules and pathways affected by both. In this respect, we anticipate that further understanding of these molecular targets will be relevant to both therapeutic and etiological aspects of ASD. Similarly, such studies will hopefully help us to understand ASD-related epigenetic modulation and developmental alterations implicated in the neural and behavioral impairments.

In Table 4, we have summarized outcomes, breakthroughs, or major findings in animal models, relating to ASD and immune system activation. In the case of animal models of maternal immune activation, there is a cascade of inflammatory responses that are dependent on the pathogenic agent and can potentiate immune responses in offspring in a strain-dependent manner (111). It is hypothesized that pro-inflammatory cytokines, brain-reactive antibodies, and endocrine mediators, such as corticotropin-releasing factor and glucocorticoids participate in the etiology of autoimmunity-associated behavioral syndrome (164). Also, neonatal rat infection with Borna disease virus results in abnormalities of early development and increase in locomotor activity; stereotypies and brain expression of mRNA for IL-1α, IL1-β, IL-6, TNF-α, and TNF-β (165).

**Table 4:** **Selected findings in animal models related to ASD and immune system**.

Model	Animal	Outcome, breakthrough or major finding	Reference
–	Mouse	Suggested that animal models of autoimmunity-associated behavioral syndrome (AABS) may be a useful model for the study of CNS involvement in human autoimmune diseases, e.g., autism	(164)
Neonatal rat infection with Borna disease virus	Rat	Abnormalities of early development; Increase locomotor activity; Increased stereotypies; Increased brain expression of mRNA for IL-1a, IL1-b, IL-6, TNF-α, and TNF-β	(165)
MIA	Mouse	Offspring display deficits in prepulse inhibition; deficiency in exploratory behavior and deficiency in social interaction	(166)
MIA	Mouse	Prepulse inhibition (PPI) and latent inhibition (LI) deficits were observed in the adult offspring. Coadministration of an anti-IL-6 antibody in the model of MIA prevented the behavioral changes. MIA in IL-6 knockout mice does not result in several of the behavioral changes seen in the offspring of wild-type mice after MIA	(167)
Prenatal exposure to VPA	Rat	Increased basal level of corticosterone, decreased weight of the thymus, decreased splenocytes proliferative response to concanavaline A, lower IFN-gamma/IL-10 ratio, and increased production of NO by peritoneal macrophages	(159)
Prenatal exposure to antibodies from mothers of children with autism	Mouse	Adult mice exposed *in utero* to IgG from mothers of children with autistic disorder displayed anxiety-like behavior and mice had alterations of sociability; evidence of cytokine and glial activation in embryonic brains	(168)
MIA	Rhesus monkey	Behavioral alterations in infants monkeys were observed, e.g., disruption of prepulse inhibition. Magnetic resonance imaging (MRI) revealed a significant 8.8% increase in global white matter volume distributed across many cortical regions compared to controls	(169)
MIA	Mouse	Pups born to maternal immune activation (MIA) mothers produce a lower rate of Ultrasonic vocalizations, decreased sociability and increased repetitive/stereotyped behavior	(170)
MIA	Mouse	Systemic deficit in CD4(+) TCRβ(+) Foxp3(+) CD25(+) T regulatory cells, increased IL 6 and IL-17 production by CD4(+) T cells, and elevated levels of peripheral Gr-1(+) cells; hematopoietic stem cells exhibit altered myeloid lineage potential and differentiation; behaviorally abnormal MA offspring that have been irradiated and transplanted with immunologically normal bone marrow from either MIA or control offspring no longer exhibit deficits in stereotyped/repetitive and anxiety-like behaviors	(110)
MIA	Rhesus monkey	Offspring exhibited abnormal responses to separation from their mothers, increased repetitive behaviors and inappropriately approaching and remaining in immediate proximity of an unfamiliar animal	(171)
Prenatal exposure to antibodies from mothers of children with autism	Mouse	Offspring displayed autistic-like stereotypical behavior in both marble burying and spontaneous grooming behaviors. Additionally, small alterations in social approach behavior were observed	(172)
MIA	Mouse	Following stimulation macrophages from offspring of poly(I:C) treated dams produced higher levels of IL-12, suggesting an increased M1 polarization. Also, macrophages from offspring of poly(I:C) treated dams exhibited a higher production of CCL3	(173)
MIA	Mouse	In the marble burying test of repetitive behavior, male offspring but not female offspring from both LPS and PolyIC-treated mothers showed increased marble burying	(174)
Prenatal exposure to VPA	Mouse	VPA mice present signs of chronic glial activation in the hippocampus and the cerebellum; When they are challenged LPS, they show an exacerbated inflammatory response, increased expression of pro-inflammatory cytokines in the spleen and higher corticosterone secretion to the blood	(112)
BTBR strain	Mouse	Levels of IgG isotypes deposited in fetal brain of BTBR mice were significantly higher than in FVB mice except for IgG1	(175)
BTBR strain	Mouse	Altered IgG levels were found, e.g., higher IgG1:IgG2a ratios; presence of brain-reactive IgG in the sera; levels of IgG1 deposited in the cerebellum, cortex, hippocampus or striatum of both BTBR male and female mice were significantly higher than in FVB counterpart	(176)
MIA	Mouse	Adult LPS-treated mice offspring had an elevated percentage of interferon (IFN)-γ(+) CD4(+) T cells and interleukin (IL)-17A(+) CD4(+) T cells in the spleen, IL-17A(+) CD4(+) T cells in the liver, and CD4(+) Foxp3(+) T cells in the spleen. LPS offspring CD4(+) T cells showed increased proliferation and an enhanced survival rate	(177)

Animal models of maternal infection have also been used to study behavioral impairments and brain alterations, such as maternal influenza infection (166), maternal immune activation (110, 167, 169–171, 173, 174, 177, 178), and prenatal exposure to antibodies (168, 172). In addition, the inbred BTBR T + tf/J (BTBR) mouse strain has been used as an animal model of core behavioral deficits in autism. BTBR mice exhibit repetitive behaviors and deficits in sociability and communication, presenting higher IgG1:IgG2a ratios and increased levels of IgG1 in brain (175, 176).

## Summary and Outlook

**Figure 2:**
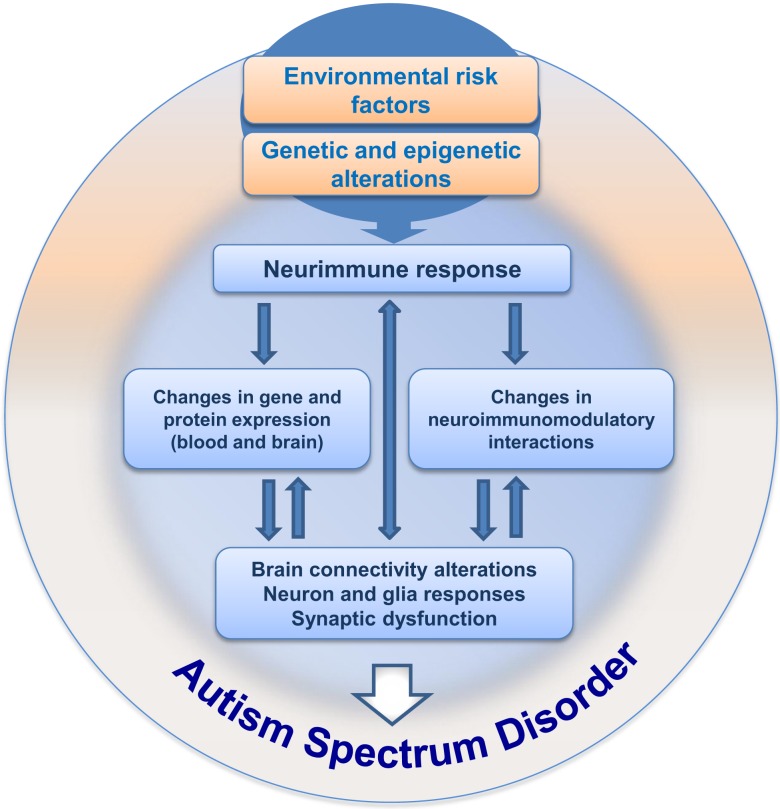
**Hypothesis for neuroimmune interactions in triggering the development of ASD**. This hypothesis considers the presence of environmental risk factors during pregnancy, followed by immunoneuroendocrine response from the mother to the developing embryo/fetus. The risk factors (such as VPA) would influence central and peripheral neural responses in the context of a crosstalk with the immune system, followed by gradual changes in neural plasticity and function, resulting in behavioral impairment during development, ultimately leading to ASD.

Since the first descriptions of autism, 70 years of investigation have passed, with great efforts mainly in the last decade, bringing important information and knowledge on the mechanisms underlying ASD. Nevertheless, even with these advances, the etiology of ASD remains largely unknown and we are still searching for specific clinical marker(s) able to improve early diagnosis. We work on the hypothesis that integrating maternal–embryo systems will contribute to the understanding of ASD. One possibility, which was summarized here, concerns the hypothesis of neuroimmune interactions being involved in triggering ASD development, as schematically depicted in Figure 2. The presence of environmental risk factors during critical periods of embryonic/fetal development may influence the immune system in the mother, promoting localized or systemic inflammatory responses with the release of cytokines and hormonal molecules, which in turn, via neuroimmunomodulatory responses and crosstalk between circulatory and neural systems, may impair circuitry development, neuronal plasticity, and neuroglial function in the embryo/fetus. As immunological factors interfere with neural development since the embryonic period, and considering that inflammation or immune response may arise due to abnormal environmental interactions *in utero*, a better understanding of the neuroimmune changes that may underlie the pathogenesis or pathophysiology of ASD will hopefully have a large impact on the development of new clinical and therapeutic strategies to better deal with ASD.

## Conflict of Interest Statement

The authors declare that the research was conducted in the absence of any commercial or financial relationships that could be construed as a potential conflict of interest.
